# Lessons From Joint Improvisation Workshops for Musicians and Robotics Engineers

**DOI:** 10.3389/frobt.2020.576702

**Published:** 2021-02-19

**Authors:** Anthonia Carter, Marianthi Papalexandri-Alexandri, Guy Hoffman

**Affiliations:** ^1^ Information Science Department, Cornell University, Ithaca, NY, United States; ^2^ Music Department, Cornell University, Ithaca, NY, United States; ^3^ Sibley School of Mechanical and Aerospace Engineering, Cornell University, Ithaca, NY, United States

**Keywords:** improvisation, robotics, artificial intelligence, thematic analysis, observational methods, workshops

## Abstract

We report on a series of workshops with musicians and robotics engineers aimed to study how human and machine improvisation can be explored through interdisciplinary design research. In the first workshop, we posed two leading questions to participants. First, what can AI and robotics learn by how improvisers think about time, space, actions, and decisions? Second, how can improvisation and musical instruments be enhanced by AI and robotics? The workshop included sessions led by the musicians, which provided an overview of the theory and practice of musical improvisation. In other sessions, AI and robotics researchers introduced AI principles to the musicians. Two smaller follow-up workshops comprised of only engineering and information science students provided an opportunity to elaborate on the principles covered in the first workshop. The workshops revealed parallels and discrepancies in the conceptualization of improvisation between musicians and engineers. These thematic differences could inform considerations for future designers of improvising robots.

## 1 Introduction

This paper describes a series of workshops that were conducted with the goal of understanding what lessons researchers in AI and robotics can draw from the practice of musical improvisation in the process of designing improvising robots and AI-enabled musical instruments. We invited professionally practicing multidisciplinary musicians with substantial improvisation experience to attend a day-long workshop together with Mechanical Engineering (ME) and Computer and Information Science (CIS) graduate students. Themes extracted from the artists’ experiences were later presented in follow-up workshops to additional CIS and ME researchers and discussed in the context of AI and robotics engineering. These investigations present a number of interrelated concepts of interest to designers aiming to build improvising machines. The investigations also revealed differences in how the conceptualization of improvising machines differs between practicing musicians and robotics engineers. These thematic gaps should be taken into account when these two populations collaborate creatively.

AI and robotics improvisation has been studied from the perspective of algorithmic structures as well as from that of cognitive models. In this work, we instead take an exploratory design-observation approach by asking professional improvising musicians and engineers to participate in a joint workshop. We started the exploration by posing a question to our workshop participants: “What is an improvising musical robot?” The motivation behind this question was to capture initial thoughts around combining AI with improvisation without encouraging bias toward any particular knowledge tradition. This opening question grounded the gathering as a joint venture between engineering and music by investigating cases for artificially intelligent improvisation agents.

During this initial workshop, four improvisation themes crystallized: Improvisation as *Spontaneity*, Improvisation as *Adaptability*, Improvisation as *Learning*, and Improvisation as having an *Inner Voice*. Improvisation as Spontaneity captures the requirement to embrace uncertainty and the element of surprise inherent in improvisation. Improvisation as Adaptability embodies the actions of an improviser when responding to environmental factors such as setting, audience, and ambience, as well as musical stimuli stemming from other members of the musical ensemble. Improvisation as Learning embodies the improviser’s ability to use past knowledge to make on-demand decisions. Improvisation as an expression of Inner Voice focuses on the improviser’s agency in producing personally distinctive content true to their conscience.

We also encouraged workshop participants to consider the role of AI and robotics in improvisation. This prompt uncovered three translative themes for AI and robotics: Improvisation as *Randomness*, Improvisation as *Assistance*, and Improvisation as *Data*. Improvisation as Randomness is the act of an artificially-intelligent agent causing disturbances during a musical performance. Improvisation as Assistance is about using AI and robotics to assist the decision-making process and provide feedback for human improvisers. Improvisation as Data highlights the fact that sound as data can be fed into a machine learning model. While these translative themes might suggest that many workshop participants see AI improvisers in a diminished role, workshop contributors also repeatedly considered AI and robotics as “superhuman,” embodying the idea of artificially intelligent agents transcending the human physical body, memory, and capacity to improvise.

We conducted two follow-up brainstorming workshops with researchers to further explore how engineering and CIS researchers conceptualize notions of improvisation based on insights from the joint workshop. The goal was to develop, explore, and solidify the ideas that came up in the first workshop. As a result of all three meetings, we argue that the themes that emerged from the musicians’ *human* experiences of improvising both contrast and match the way that the group conceptualized *machine* improvisation. Our findings point to considerations that may be useful to designers of improvising robots trying to bridge the engineering and musical improvisation communities.

## 2 Theoretical Background

The themes explored in the workshops can be viewed against a background of literature from cognitive models of human improvisation in non-artistic realms on the one hand (as cognitive models often inspire AI and robotics computational models), and from theories and practices in artistic improvisation on the other hand.

### 2.1 Cognitive Models of Improvisation

Cognitive scientists have long identified the human ability to improvise as central to our problem-solving processes, and in particular the ability to create previously unknown solutions to existing problems. They see its importance particularly when it comes to real-time decision-making under time pressure (e.g., Moorman and Miner, 1998; Mendonca and Wallace, 2007).

#### 2.1.1 Temporal Convergence During Environmental Turbulence

Mendonca and Wallace (2007) conceptualize improvisation as the temporal *convergence of planning and execution*. This definition of convergence is consistent with empirical accounts, from disaster response, through medical diagnosis and treatment, to sports. Analysis of these scenarios define improvisation as a situation in which thinking and acting, or reading and reacting, come together (e.g., Irby, 1992; Bjurwill, 1993). Moorman and Miner’s analysis focuses on product development, and finds that improvisation occurs when an action is required, no plan is in place for the situation encountered. Improvisation events are most frequent when there is increased “environmental turbulence,” a situation where information is flowing faster than it can be processed. In these cases, agents draw on short-term cues to make decisions, rather than integrate long-term prior information. These findings point to the fact that improvisation could be useful in areas of anticipated robot deployment, such as in medical or emergency response scenarios. Empirical evidence in both Mendonca and Wallace and Moorman and Miner shows that in those situations of turbulence, improvisation can result in better outcomes than planning. However, their detailed analysis also finds that long-term knowledge that existed prior to the improvised event positively affects improvisation outcomes. These findings suggest that a combination of learning, spontaneity, and adaptability are central to successful improvisation in dynamic settings.

#### 2.1.2 Problem Solving, Planning, and Re-Planning

A different cognitive view of improvisation comes through the lens of problem-solving (Newell and Simon, 1972), where agents navigate from an existing state to a goal state. The above-stated situations of reacting to environmental turbulence can then be thought of as the addition and deletion of problem states, as well as the reclassification of the current state, or the redefinition of states as goal states. The planning problem of finding paths through the problem state is thus transformed into finding new paths through the problem state space (Ramalho et al., 1999). If this happens under time pressure, improvisation may be necessary. An additional cognitive problem facing an improvising agent is knowing *when* to diverge from an existing plan. This can be in response to time pressure or when it is unlikely that reasoning can result in an appropriate action (Mendonca and Wallace, 2007). Improvising agents also need to make comparisons between similar action and state paths in order to correctly categorize alternative paths of action (Horty and Pollack, 2001).

#### 2.1.3 Slow Monitoring and Fast Reactive Control

From a computational modeling perspective, researchers have suggested that improvisation relies on a two-stage model of activity, including a slow process that monitors and evaluates the action of an agent and compares it to incoming feedback, and a faster one running as an open-loop motor program which cannot be interrupted (Glencross, 1977; Pressing, 1984). Training and learning in these frameworks are modeled as the progressive decrease of cognitive load by offloading motor programs to open-loop memory. Researchers have also offered models of opportunistic planning and of two-step decision-making systems in which the improviser first uses long-term memory to select a subset of appropriate actions and then uses rapid decision-making based on instantaneous feedback to select among those actions (Hayes-Roth et al., 1997; Rousseau and Hayes-Roth, 1998).

#### 2.1.4 Declarative and Procedural Knowledge

Mendonca and Wallace (2007) offer a cognitive model which includes an ontology for declarative knowledge and a decision logic for procedural knowledge, both of which the agent possesses. Their framework includes processes that can compare planned routines and alternative action sequences and can produce mappings of interchangeable resources to be used in real-time for opportunistic action. Canonne and Garnier (2011) emphasize additional useful concepts in their cognitive model of improvisation. These include considering a gradient of time scales in decision-making, from fractions of seconds to several minutes. They tie this into a model of information processing which models the dynamics of an agent’s objective and intentions. Finally, they account for cognitive load and boredom for when action replacements need to occur.

In summary, researchers in a variety of fields have proposed cognitive models for when and how people improvise. Some common themes are the operation on multiple time scales, the temporal convergence of learning and acting, and the ability to act based on new information. Different types of knowledge are acknowledged, indicating that improvisation is never a completely reactive skill, but that prior knowledge is also not enough to make decisions in the moment. These models provide a fertile ground for exploring the possibility of a computational cognitive framework for AI and robotics improvisation.

### 2.2 Theories, Methods, and Practices in Performance Art Improvisation

While most humans improvise in their daily lives, professional performers often study and practice improvisation in a structured way. Learning from their methods can help gain a better understanding of the systematic constructs of the improvisational process. This section provides a brief study of some of the theories, methods, and practices for improvisation documented by performing artists that could serve as a basis for a computational cognitive framework of artificial improvisation.

#### 2.2.1 Referent Motifs and Variations on a Theme

A recurring concept in performance improvisation is the “referent” (Pressing, 1984; Magerko et al., 2009). In music, for example, this is often a melodic theme or motif. The improvisation’s relationship to that referent can then take one of several forms: the ornamentation of the referent, a variation of the referent, or a temporal synchronization with the referent. Some authors emphasize that divergence from the referent opens the possibility for new structures (Klemp et al., 2008). The referent’s origin can also be one of a number of sources. It can draw from a commonly accepted canon or from an instantaneously perceived event, such as an action done by another artist. In translation to human-robot interaction, this relationship could be modeled as 1) an action filling in an incomplete plan, or meshing the robot’s action to the current human action sequence (ornamentation), 2) an alteration of an existing plan, either from an offline database or based on currently perceived human action sequences, or 3) time-warping an existing plan to match the action sequence of the human.

#### 2.2.2 Object Memory and Process Memory

Looking at the training of improvisers, we often see the repeated performance of referents alongside variations on the referent. This practice enables the improviser to learn two distinct things: One is the “object memory” of the referent, building up a database of canonical knowledge. The other is “process memory,” which teaches the agent schema of compositional problem-solving such as: variations, transitions, and so forth (Pressing, 1984). This division into two types of memory relates to the previously mentioned insight that improvisation occurs at two time scales. The referent evolves slowly, with little decision making, but with continuous feedback and monitoring. The short-term decision-making process works locally and on a shorter time-scale, using “process memory” such as variations in an open-loop fashion which enable it to act quickly. Acting on the slower time scales is thus often piece-specific, whereas acting on the faster time scales is training-specific. These concepts also map in ways useful to robot planning and control, and relate to the formalisms of “declarative knowledge” and “procedural knowledge” implemented in existing cognitive architectures (e.g., Anderson, 1996).

#### 2.2.3 Mutual Responsiveness and Adaptation

An additional recurring theme is that of mutual responsiveness. Training manuals for actors and performing artists often emphasize improvisation games where actors need to react quickly to unexpected input from others. This is most strongly associated with Meisner’s “repetition exercise” (Meisner and Longwell, 1987), which states that “acting [is] responding truthfully to the other person”. Similarly, Maleczech speaks of *repercussion*: “The other actors are, for me, like the bumpers in a pinball machine. Often the next image will come directly from the response of the other actor” (in Sonenberg, 1996). Moore adds that “ensemble work means continuous inner and external reaction to each other” (Moore, 1968).

#### 2.2.4 Embodied Improvisation

Finally, many texts on improvisation emphasize the embodied aspect of improvisation. Boal states that “the human being is a unity, an indivisible whole […] one’s physical and psychic apparatuses are completely inseparable […] bodily movement ‘is’ a thought and a thought expresses itself in corporeal form.” (Boal, 2002). Other texts also emphasize the physical aspect of improvisation (e.g., Moore, 1968; Cole and Chinoy, 1970; Broadhurst, 2004). Even texts that take a symbolic and algorithmic view of improvisation note that the embodied nature of the act is central (Johnson-Laird, 2002).

To summarize, an analysis of improvisation methods in the performing arts, theories, and practices can inform the proposed computational framework. Themes such as embodiment, mutual responsiveness, and chance relationships to a referent are examples of insights to be gained from exploring the performing arts and their practices.

### 2.3 Related Work: Computational Models and Systems of Improvisation

There have been many efforts over the past decades to translate improvisation principles to computational agents, both for the sake of staging human-machine improvisation systems and to understand improvisation practices in order to build non-performative AI systems. Two prominent, and well studied, examples are the *Voyager* system developed by George Lewis and the *OMax* system coming out of the Institute for Research and Coordination in Acoustics/Music (IRCAM).

#### 2.3.1 Machine Improvisation as Inquiry

*Voyager* is an artificial improvisation system designed in the 1980s to engage with musicians on stage (Lewis, 2000). The system analyzes human performance and generates compositions in real-time. In Lewis’s analysis of Voyager, he emphasizes its role as a non-hierarchical system that “does not function as an instrument to be controlled by a performer,” but instead “emanates from both the computers and the humans.” (Lewis, 2000). The flexibility made available by this type of composition and improvisation system challenges existing notions of composed vs. improvised music and, “deals with the nature of music.” (Lewis, 2018) More recently, Lewis maps several reasons for the pursuit of machine improvisation, including the possibility to challenge traditional notions of interactivity, as well as larger societal questions of agency and choice (Lewis, 2018).

Similarly, *Odessa* is another example of a computational agent used to understand musical improvisation activities. Odessa is an artificially-intelligent agent used for cognitive modeling of human-interactive musical behavior (Linson et al., 2015). The model was used to evaluate a collaborative musical improviser through subsumption, a robotics architecture.

#### 2.3.2 Statistical Learning at Two Scales

Developed in the mid-2000s, *OMax* is a real-time improvisation system that performs style-learning from human musicians in real time, and can respond via an improvisation-generation that includes metrical and harmoic alignment (Assayag et al., 2006). OMax is grounded in statistical sequence models, taking into account both short-term and long-term sequences, thus operating on two time scales. It also includes a multi-agent architecture, enabling it to instantiate different improvising agents into compositional topologies. The system is primarily focused on the analysis, learning, and construction of musical elements. In a recent dissertation, Nika elaborates on the OMax system, emphasizing the themes of *intentions*, *anticipations*, *playing*, and *practicing* (Nika, 2016).

#### 2.3.3 Robot Improvisation as Embodied Opportunism

One of the authors of this paper developed a gesture-centric robot improvisation system (Hoffman and Weinberg, 2011). In contrast to previous works, the system was not structured around analysis and response, but instead used the robot’s physicality and spatial movements to react in real time to musical impulses. This system highlighted several of the above themes, including operating on two time scales and on anticipation, but also it contributed the notion of *opportunism* to musical improvisation.

#### 2.3.4 Themes for Machines Improvisers

In an effort to extract improvisation themes for computational agents, Magerko et al. (2009) videotaped professionals performing improvisation games and used a retrospective think-aloud protocol to extract themes. They found that improvisers use a number of cognitive processes, including inference from others’ actions, narrative development, and referent use. However, they state that it “is not yet clear is how these different findings can be synthesized into a more singular, comprehensive viewpoint.” The authors used insights from these workshops to build an improvising virtual reality agent (Hodhod et al., 2012). Their approach is grounded in logic and is embodied in a virtual character rather than an embodied robot.

Mogensen (2019) analyzes two Human-machine improvisation systems (Voyager, which is mentioned above, and *Favoleggiatori 2* Mogensen (2018)) from the perspective of Soft Systems. He describes such systems as a “nonverbal exchange between human and computer,” using elements such as *memory*, *motivations*, *values*, and *search strategies*.

In a recent paper, Lösel (2018) surveys this work along with other research on artificial musical improvisation to suggest the following points of focus for computational models of improvisation: *embodiment*, or using the physical space and body of the agent for the improvisation process; and *chance and emergence*, suggesting that there should be a process in place which enables divergence from planning so that actions can be created. This work, however, has not been developed into a working computational system. Most recently, Kang et al. (2018) explored improvisation in the context of collaborative art practice and as a research lens for human-computer interaction, and also identified the following key themes: *reflexivity*, *transgression*, *tension*, *listening*, and *interdependence*.

In summary, researchers have long explored themes of improvisation in the context of machine improvisation, as well as in the context of computational models of human improvisation. Most of these works were developed with music in mind, and centered around disembodied musical agents, although some have also considered embodiment and robotics. The currently presented study does not propose to contribute to the rich theoretical literature in the field, but is instead aimed at exploring how the viewpoints of practicing musicians and robotics engineers engage with each other when they are placed in the context of a design activity to develop an improvising machine.

## 3 Joint Workshop

We began by conducting a full-day workshop that brought together two groups of experts related to the topic of robot improvisation. The first group were members of a professional musical ensemble with extensive improvisation experience on and off the stage. The second group were graduate and undergraduate students in Music, Mechanical Engineering (ME), Computing and Information science (CIS), and Design departments. The musicians were part of an ensemble that has previously collaborated with one of the authors (Papalexandri-Alexandri), and with one of the music students in the workshop. They helped invite students from a local music academy.

We also recruited a select group of ME and CIS graduate students in robotics labs across the authors’ university to participate in a one-day workshop on AI, robotics, and improvisation. Students submitted a short, one-paragraph statement describing their relevant background experience and interest in the workshop. We received nine statements, five from graduate students in ME CIS, design, and four undergraduate students in music. Ultimately, seven of nine applicants participated in the joint workshop. Other guests included workshop organizers from the music department at our university and administrative staff from the local music academy.

Our motivation for inviting this group was to bring together diverse perspectives on improvisation and artificial intelligence to speculate potential futures for AI improvisation models. There were a total of sixteen collaborators in the workshop. The core of the workshop was structured around two roughly equal parts that addressed AI and robotics (part I) on the one hand, and musical expression (part II) on the other and was comprised of lectures, open discussions, technology demonstrations, and musical games.

**FIGURE 1:**
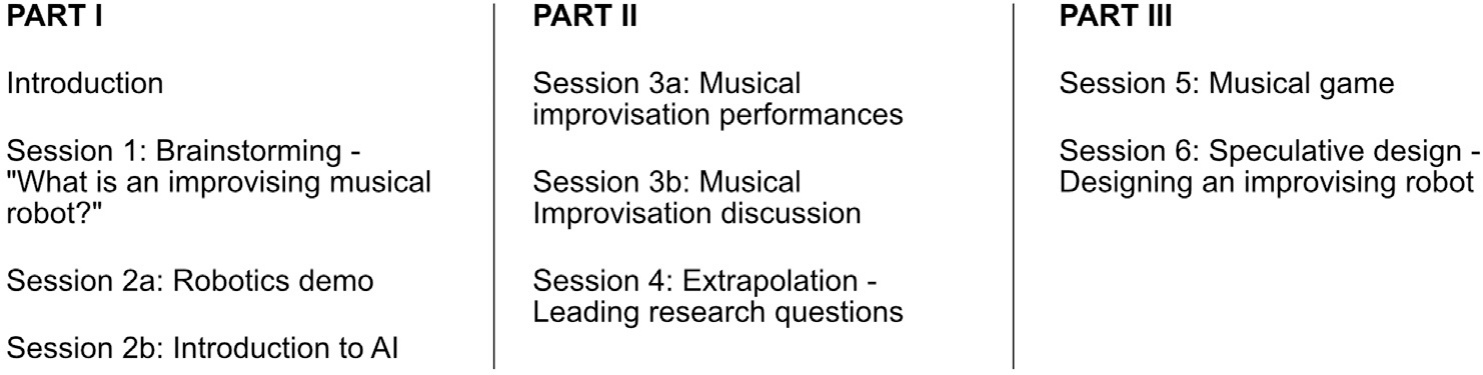
The first workshop was structured six sessions, divided into three parts. The first covered mostly robotics and AI, the second covered musical improvisation, and the third was more exploratory. Both Part I and Part II also had an exploratory element (Sessions 1 and 4).

The schedule was structured as follows, depicted in Figure 1: Session 1, a warm-up brainstorming activity, Session 2, an AI and robotics demonstration, a lecture and subsequent discussion, Session 3, musical improvisation performances and a subsequent discussion, Session 4, a written response extrapolation activity, Session 5, a musical game, and Session 6, a speculative design activity.

After introductions, the first activity was a warm-up exercise in which we asked workshop participants: “What is an improvising musical robot?” We wanted to capture initial thoughts on combining AI with musical improvisation before introducing insights or principles from both fields. We gave everyone 5 minutes to write down ideas that came to mind. At the end of the activity, we collected ideas ranging from an AI system capable of adjusting musical parameters based on real-time input to robots bringing a distinctive sound, voice, or resonance to a musical setting. These themes would be echoed later throughout the day’s workshop.

**FIGURE 2:**
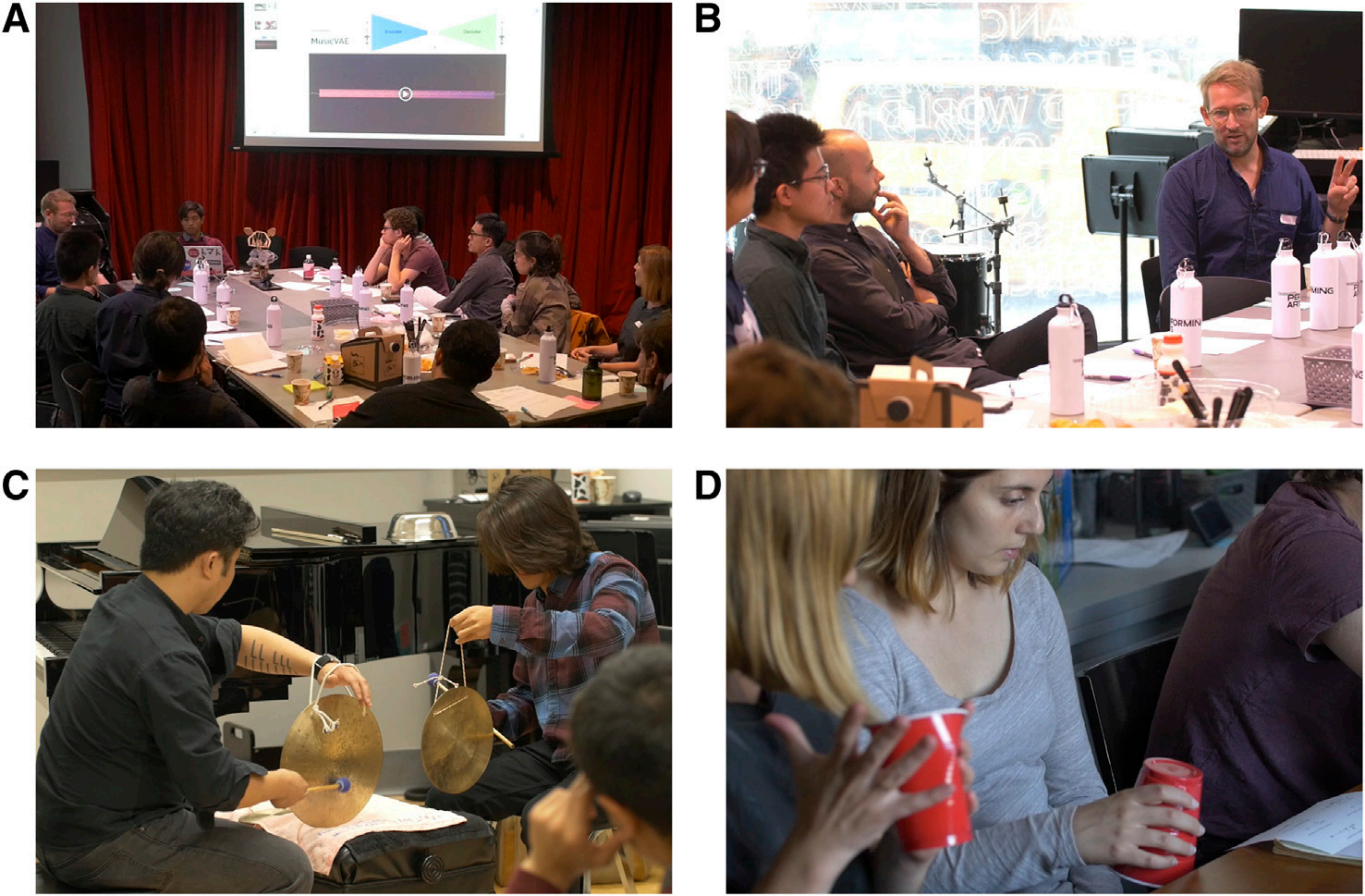
Four of the sessions conducted in the first workshop: **(A)** Robotics demonstration (Session 2a), **(B)** AI and robotics introduction and discussion (Session 2b) **(C)** Musical improvisation performance (Session 3a), and **(D)** Musical games (Session 5).

The second activity included a robotics demonstration and a brief introductory lecture on AI and robotics (Figures 2A,B). The first part of the lecture gave a brief history of AI in the context of relating human intelligence to utility theory and logic. Next, a graduate ME student presented a demonstration in which an AI system was used to generate musical notes and gestures performed by Blossom, a social robot (Suguitan and Hoffman, 2019). The intention of the demonstration was to present AI and robotics to the musicians in the workshop in a tangible manner. The choice of demonstration was also intended to connect with a specific application of deep learning relevant to musicianship. As to be expected in the introduction of a new topic, much of the discussion was about the mechanics of the neural network underlying the demonstration. Musicians were curious, for example, about how an AI system compresses and decompresses music samples into and from a lower-dimensional space. There was also discussion about how musical applications of AI differ from other AI applications such as board games.

The third activity was a series of musical improvisation performances followed with a discussion debriefing the performances (Figure 2C). The session was structured around three musical improvisation duos. The first was a percussion duo using gongs. The musicians traded rhythmic and timbre phrases and used the gongs in a variety of physical configurations, such as hanging, lying on the table, and so on. The second performance was by a string duo that included a violin and an electric guitar. The guitarist made heavy use of electronic filtering and amplification devices, often using the noise generated by the devices as musical elements. The violinist also went beyond the classical use of the instrument, i.e., bowing and plucking, and improvising not only with the musical notes, but also with the physical use of their instrument. The third duo was of wind instruments, which included a flute and a trombone. Again, the flutist did not produce sounds in the classical way, but instead produced a variety of voiceless breathing sounds through the flute. The trombonist similarly used the instrument as both a brass instrument and as a percussion surface. Each performance lasted about 5–7 minutes. In each duo, one musician would emerge in the role of a leader with the other as their follower. The leader would set a tone, and the follower would either complement or contrast the sound. At times, the playing would converge before drifting apart again. In some cases, it seemed that the leader-follower roles were predefined or at least implicitly understood. In others, the roles seem to emerge organically from the improvisational interaction.

The subsequent discussion was seeded with the reflections of the musicians. The opening question was: “From a personal [experiential] perspective, what does it mean to improvise?” Responding to this question, musicians noted that improvisation is about being open to the environment around you, including other musicians. One musician described it like a conversation in which you need to communicate to your partner that you are open to what they have to “say.” Musicians generally describe improvisation as a very specific type of intelligence (Matare, 2009). In contrast to scored music, one has to often choose between a huge number of possible actions in improvisation. Some musicians described it as the art of managing musical or logistical constraints and imposing or eliminating self-censorship while operating in a state of vulnerability. In line with this vulnerability, many also described improvisation as a risky activity. We will discuss many of these themes in the following sections.

In the fourth activity, all workshop participants individually responded to two leading research questions: “What can AI and robotics learn by how improvisers think about time, space, actions, and decisions?” and “How can improvisation and musical instruments be enhanced by AI and robotics?” Participants were given 10 minutes to respond to the two questions. They were first asked for written responses, and then one by one, each person shared aloud their responses to each question. Most commented on a robot’s ability to go beyond the physical limitations of human capacity. An AI system can pursue multiple paths forward at any given moment, thus narrowing the space between intent and action. These themes are also discussed in detail below.

The fifth activity was a music improvisation game led by a graduate student in the field of music composition. Workshop participants used plastic cups to produce sounds and explore possibilities of *sound material* by transforming ordinary objects into instruments. Players were asked to improvise in several segments, inspired in turn by the concepts of “Being Human,” “Being Machine,” and “Being Intelligence” (Figure 2D). The improvisation was guided by a musical score, but players were encouraged to break from the score and interpret it as they saw fit. In *Being Human*, performers focused on creating music “with the consciousness of a human.” In *Being Machine*, performers experimented with the sound and knowledge of the instrumental arrangement, driven by more mechanistic patterns and motivations. Finally, in *Being Intelligence*, performers created scenarios where they become machines with intelligence. Each rhythm produced by the players combined into a musical pattern, which was performed simultaneously by the full group of participants. This process led each person to make decisions about how to proceed playing based on the collective sound of the group. The experience created several forms of sound production and musical “algorithms” in terms of sound patterns and helped provide an embodied experience of both improvisation and algorithmic thinking.

The workshop ended with a speculative design activity for creating an improvising musical robot and a final show and tell. We split up into four groups and used this session as an opportunity to form multidisciplinary groups of musicians and engineers to produce ideas for an AI improvising agent. Workshop participants were encouraged to reflect on the day’s activities and discussions and integrate them into a specific design. The outputs of the exercise were four designs that ranged from musical devices to an adversarial performance venue. The detailed descriptions of these designs are beyond the scope of this paper.

## 4 Improvisation Themes From the Joint Workshop

During the joint workshop, several recurring themes emerged. To better reflect on the activities, we collected audio recordings of the above-listed sessions. In total, there were 113 minutes of recording from the one-day workshop split across five audio fragments. We then used a combination of automated transcriptions and audio review of these recordings to retrieve and collate these themes through affinity diagramming. We assigned tags at time points that corresponded to quotes from each of the themes. Themes were elucidated and refined through an iterative analysis process and consensus-building discussion among the authors.

By nature of their profession, the practicing musicians who participated in the workshop had a significantly longer life experience with improvisation than the other participants. As a result, the musicians contributed a larger body of data, and many of the discussion themes are grounded in their comments. Summarizing our insights from the audio transcriptions, we identify an overarching description of improvisation as the ability to generate new material in real time and of one’s own accord while paying attention to or being influenced by one’s surrounding environment. This ability, however, is not developed in the moment alone, but is based on *extensive prior practice* and knowledge of predominant motifs. The above definition contains four themes: Improvisation as *Spontaneity*, Improvisation as *Adaptability*, Improvisation as *Learning*, and Improvisation as an expression of an *Inner Voice*.

The majority of the data supporting each theme came from the 45-minutes improvisation discussion with the practicing musicians. For Improvisation as Learning, one quote came from the extrapolation activity. The themes mentioned above sufficiently capture the musical improvisation discussion’s viewpoints, and none were broken into sub-themes.

### 4.1 Improvisation as Spontaneity

Improvisation as Spontaneity is the ability to generate *new and often surprising material in real time*. The real-time component of improvising steers improvisers to make choices about how to produce sound on the spot. As musicians, do not have an abundance of time to deliberate on what note to play next or anticipate how the overall tone of the performance will be when improvising, there is a degree of urgency more present in improvisation performances and less present in rehearsed performances.

When asked about the relationship between the past, present, and future in an improvisational setting, Workshop Participant 1 (WP1), a trombonist, stated that they wanted “to narrow that gap or eliminate it as much as possible so there is no future or past and that means bringing the awareness to the current sonic world.” In closing this gap, one is fully cognizant of the immediate moment in time. This ties back to the view of improvisation as the temporal convergence of thinking and acting as stated by Moorman and Miner (1998). WP6 (pianist) describes anything improvised as having “a real-time component [where] actions are thought [of] and produced in the moment.” It is at this critical moment where intention meets action.

Similarly, according to WP1 (trombonist), mastery of improvisation is “bring[ing] the intent up to the action,” so that “there is no time delay” between thought and action; the strategy and implementation is almost instantaneous. This mode of being was described by WP5 (violist) as getting into “this space that feels like above your head,” or being “in another zone” where “things are just happening.” This points to a sublime experience associated with the spontaneous nature of improvisation.

Subsequent group discussions, however, introduced more subtlety to this theme. Spontaneity is not absolute, nor completely unrooted, but harkens back to existing knowledge, calling to mind the notions of procedural and declarative knowledge (Anderson, 1996). The musicians spoke about different levels of improvisation. WP6 (pianist) described it as follows: “There is one layer, free improvisation,” where “everything is free,” you play “with people you have never met.” On the other hand, in many Jazz improvisation setups, musicians “practice ten thousand licks in all twelve keys, and then [they] go to a session and just play one of them.” The degree of improvisation is determined by the number of free parameters available to manipulate while performing, but these are also a fluid aspect of the specific improvisation setting. This brings to the fore the complex relationship between spontaneity, pre-determined rules, and knowledge.

### 4.2 Improvisation as Adaptability

Improvisation as Adaptability is the ability to *pay attention and respond to one’s surroundings* while producing material. It embodies the actions of an improviser when responding to environmental factors like setting, audience, or other members of the band and inherently requires giving up control. WP1 (trombonist) characterizes this process as being “subordinate to what the space needs sonically.”

Improvisation implies an openness or willingness to be influenced by a surrounding environment. WP5 (violist), describes improvisation as “placing [your musical voice] in a state of openness or vulnerability so that external factors” influence “your voice and your experience.” You are “allowing your voice to change in response.” This can be being open to other musicians but also “playing solo, it could be [the] audience in the room affecting what you are doing.” This point relates to mutual responsiveness in “repetition exercise” where actors truthfully respond to each other (Meisner and Longwell, 1987). However, “your perception of how the audience is feeling might affect what you [are] doing” (WP5, violist).

When improvising, the act of creation is intertwined within the context of the surrounding space. In this contextualization process, an improviser is continuously reading and interpreting the environment or the ambiance of the room to pick up on cues on when to shift gears, such as when to adjust tone, timbre, pitch, or sound. WP3 (guitarist) explains this process as follows: “I went into [the performance] with an idea […] but my direction completely changed 5 seconds into it because [I was] making music or creating sound with another person.” These signals may be as overt as an extended deep inhalation or as subtle as a nod from an improvising partner. Such signals are essential for collaborative interactions such as improvisational performances. Sometimes however, these signals are lost in translation. WP9 (percussionist) reflects on their duo performance with WP1 (percussionist) and on having missed their partner’s intention: “If I had been able to realize the form [understand the structure of what the other person was playing], my ideas would have been completely different. […] My ideas could have complemented that form.”

### 4.3 Improvisation as Learning

Improvisation as Learning is the ability to generate material while not only paying attention to one’s surrounding environment but also to build on the improviser’s ability to use *prior knowledge acquired through study* or experience. The improviser must decide how to synthesize or incorporate discoveries into the creation of new sound material. Improvisation as Learning relates to three concepts: practice and feedback, exposure, and trust.

Building one’s musical proficiency through practice and formal training can give an improviser a range of options to pull from when performing. In the training phase, improvisers frequently perform referents to learn object memory of the referent and process memory, which teaches compositional problem-solving (Pressing, 1984). WP6 (pianist) explains that “the more you know an instrument, the more options you have. [A] top-level percussionist [knows] a thousand ways to tap a surface,” but a novice may only know a few ways to do so. “You can practice a specific way of playing a phrase or note” to develop proficiency. Still, the way “you contextualize” the learning “in the moment” is the determining factor of true skill as an improviser. On the other hand, not knowing an instrument proficiently could also be a blessing, as you have no preconceptions about how it should be played and or used. Too much knowledge can make the performer overly conscious about choices, causing them to rely more on their own and others’ expectations. In this sense, a machine has an advantage by not having too much prior knowledge.

Mastery of any skill, including improvisation, requires a mechanism for feedback or an evaluation metric. The feedback loop enables an improviser to identify areas of improvement. An experienced improviser can balance mastery of the craft with the ability to contextualize the elements surrounding the performance.

Learning through exposure is discovering new ways of working or performing outside of one’s traditional practices. WP10 (trumpeter) explains that “from a theoretical standpoint, you [do not] have to be a musician to improvise,” but practically “people generally learn [the] basics and expand from there.” In expanding a musical palette, WP2 (percussionist) says, “for me, as a musician, part of my training is I just listen to as much music as I can. I wish I had a better memory of these things […] in the fabric of [my] musicianship.”

Trust is essential in respecting other points of view or style of improvisation and having the willingness to adapt to disruptions or disturbances. It is also a process of transferring knowledge from one improviser to the next. In the workshop discussions, we learned that this knowledge of improvisation is tacit, and the sharing process is implicit and difficult to explicate. WP9 (percussionist) describes improvisation as a conversation between two people where there is “a certain continuity” or “mutual understanding that I am following. I am listening.”

### 4.4 Improvisation as an Expression of an Inner Voice

Finally, the theme of Inner Voice relates to the ability to generate material *of one’s own accord*. It stands in contrast to the previous themes, as it focuses on the improviser’s agency in producing a distinctive sound, voice, or position. In that sense, it is rooted (and not necessarily spontaneous), it is one’s own (and not necessarily responsive), and it is original (and not merely repeating prior knowledge). Inner Voice is about “bringing one’s own distinctive sound, voice, and timbre to the musical setting” (WP5, violinist). This inner voice can be a product of past experiences leading to a unique characteristic or “essence” of an improviser. WP3 (percussionist) reflects after playing a duet performance: At first, “we [did not] agree on who would lead or follow […] At that moment, I asserted a rhythmic form.” In other cases, “there are a lot of recordings where [notable improvisers] do their own thing.”

The first aspect of improvisation as having an Inner Voice focuses on how an improviser processes and synthesizes learning from experience. It involves intentionality in selecting which parts of past experiences to use, and others to disregard. This process is filtered through one’s perception and interpretation of a given situation. This filtering process is one way to develop a style of improvisation. WP9 (percussionist) performed a duet with WP3 and recalled that “there were moments where one of us decided to do something different from the other to create [a] contrast that might open the options” for different sounds. This process follows the idea of divergence from the referent noted by Pressing (1984) and Klemp et al. (2008), a mechanism to open up the possibilities for new forms to emerge.

In viewing consciousness as one’s experience, consciousness becomes the peculiar characteristic of a person, making them produce a singular sound based on a unique inner voice. Improvisation as an expression of an Inner Voice might also be one’s baggage or limited point of view (frame of reference). As WP3 (guitarist) describes it, “a lot of improvisation comes from your autopilot features” or “musical baggage,” which consists of technical skills or experience. Personal bias left unchecked might prevent one from letting external factors influence their sound and comply with these influences, thus compromising the principle of Improvisation as Adaptability.

## 5 Translative Improvisation Themes From the Joint Workshop

One of the defining features of the workshop was the convergence of thinkers from different fields. All workshop participants were encouraged to not only think about improvisation per se, but also the relationship between improvisation, robotics, and AI. We asked participants to also consider the role of an artificially intelligent machine or instrument in musical improvisation. As a result, during the extrapolation session, we uncovered “translations” of improvisation concepts into machine learning, engineering, and information science terms. We derive three translative themes which partially summarize 36 minutes of collective reflections from the extrapolation session.

### 5.1 Improvisation as Randomness

Instead of “spontaneity,” when speaking of an improvising machine, the term “randomness” often came up in workshop discussions. Improvisation as Randomness was collectively thought of as the act of causing disturbances during a musical performance. It could manifest in the form of unexpected behavior from a robotic agent on stage due to the interaction between the robot’s programmed task, physical form, and environment (Nehmzow, 2006). A robot can exhibit random behavior if any outside influence prevents it from completing a task, alters its physical structure, or changes its environment. For example, a robot could collide with the environment and break. Alternatively, sensor aliasing could result in “random” outcomes.

Randomness can also be used to take away control from a musician and to add risk. WP12 (design student) suggested that AI and robotics be used to “initialize or add more risk to the situation.” It might “contribute a specific type of sound at the very beginning” of a performance to challenge performers to interact with a distinct sound. A musician could play a robot as an instrument, and it could resist in some way. In this scenario, the musician does not have complete control over the instrument. There could be “dynamic constraints in the instruments” that affect a musician’s ability to play with another performer (WP13, CIS student).

A0lternatively, WP6 (pianist) suggests that AI and robotics “could learn a lot about human unpredictability or predictability, and we [improvisers] could enhance our improvisation practices [by collecting] data on how we form actions.” A model might anticipate a person’s next steps and create a diversion to reduce predictability by learning these patterns.

### 5.2 Improvisation as Assistance

Rather than thinking about a more egalitarian concept of “mutual responsiveness,” the idea of an improvising machine “assisting” a human musician was prevalent. Improvisation as Assistance envisions using AI and robotics to assist the decision-making process and provide feedback for improvisers. Improvisation is a continuous activity of producing thoughts and actions. Conceptualizing the bridge between thought and action as decision points, this ongoing decision-making process can be mentally exhausting due to information overload. An AI system or a robot could assist in supporting these decision points.

For example, AI and robotics could help broaden the realm of possibilities for a human improviser. They could be used to expose a user to a diverse set of music samples to help widen their scope. The repository could act as a central hub for inspiration for those looking to build on their practice, where “one idea [could be] a seed for a lot of different ideas” (WP8, saxophonist). The model can then infer a musician’s preference for a specific type of sound. Instead of suggesting similar-sounding output, the system might purposefully suggest different sounds to boost diversity and avoid over-specification (Kunaver and Požrl, 2017).

A converse way AI could assist in improvisation is through narrowing the scope of an improvisation opportunity, a critical component of the creative process. Given the spontaneous nature of improvisation, narrowing the scope of opportunity might be difficult and an improvisation space that is “too open” may make decision-making difficult. To quote one of our participants: sometimes, “musicians […] think of multiple paths forward at any given moment, but can only choose one” (WP8, saxophonist). An AI system could steer this choice for the musician. Alternatively, a user can input all of these ideas into a system that synthesizes the data and identifies patterns that can help them move forward.

Finally, AI and robotics as evaluation models might be valuable to musical improvisers. We learned that musicians’ overall consensus is they use their intuition when evaluating their performances. Evaluation models could provide a more objective feedback mechanism for improvisers, where performance metrics are supplied after each improvisation session. WP7 (Percussionist) imagined a network of sound models that could produce “a system of feedback in a digital audio workstation or the instrument” and work as an “unoriginality meter or appropriation meter.” This could encourage musicians to explore new techniques and not get stuck on old habits. The tension between the musician’s subjective sense of the improvisation’s originality and the objective metrics might in itself support a performative quality.

### 5.3 Improvisation as Data

In contrast to “learning,” workshop participants thought about “data” when considering improvising machines. Improvisation as Data highlights that sound is data and can be fed into a machine learning model. WP7 (percussionist) imagines “all acoustic instruments [as] data collection and analysis tools.” In this scenario, instruments are input devices equipped with sensors and become a “database library for the AI [model]” (WP14, music student). The initial sound data could be “enhanced to create a differentiation” of the original sound. WP15 (ME student) explains how a processing phase could be a model for learning different features. For example, “it could change the pitch of a piece or note density by looking at the structure of the data.”

Once data is fed into a model, pattern extraction can occur. The model could then begin making inferences about specific music genres. Sound data with labels such as pitch, tone, and timbre can be used in supervised learning settings to make predictions. Recall that learning improvisation occurs both internally and externally. A single person’s collective improvisation experiences are shaped by absorbing other examples of improvisation and practicing improvisation. These collective experiences can be inputs for a model that feeds “the machine of [memory] of other music and data” (P2, percussionist). The internal and external data can be parsed to help musicians make decisions as they improvise.

## 6 AI and Robotics as “Superhumans”

**FIGURE 3:**
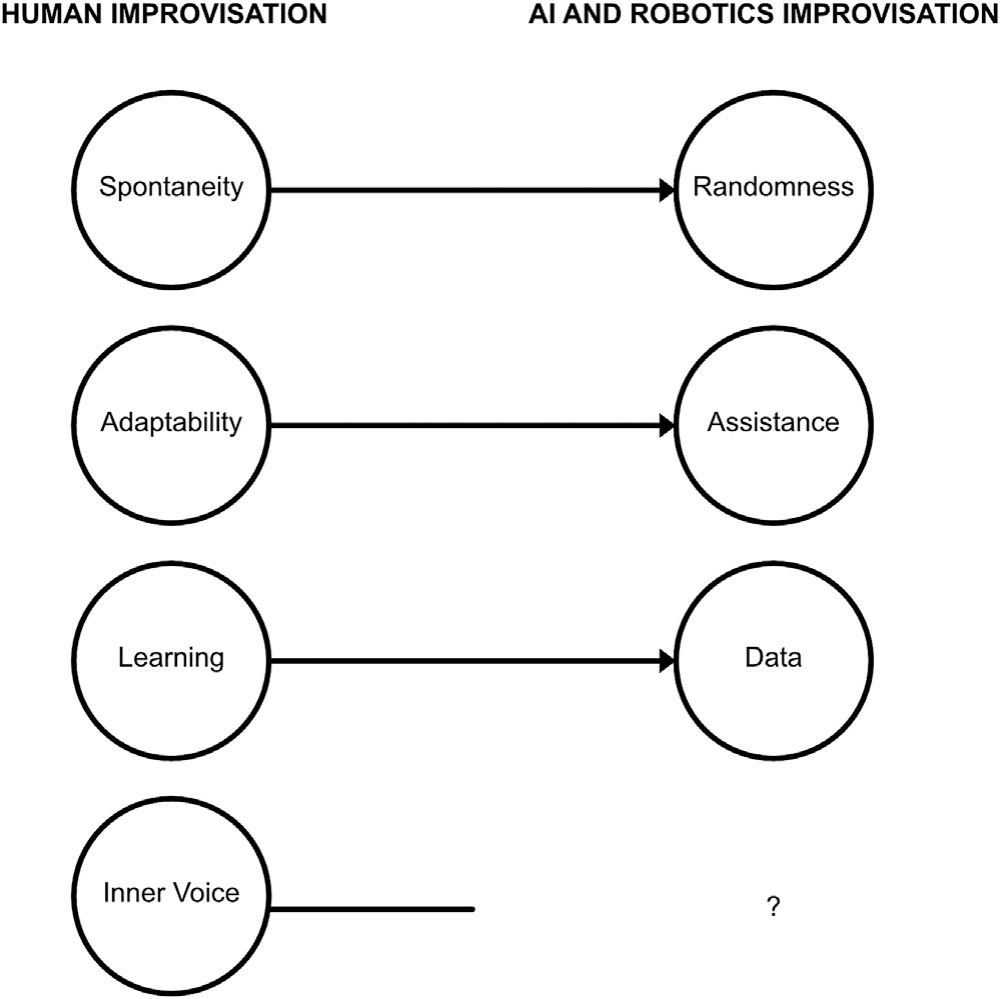
Spontaneity, Adaptability, and Learning map onto the three translative improvisation themes for machines: Randomness, Assistance, and Data. The theme of Inner Voice is missing from machine improvisation.

A closer look at the translative themes (Figure 3) suggests that AI and robot improvisers would be diminished versions of human improvisers (randomness vs. spontaneity, assistance vs. adaptability, data vs. learning, no inner voice). Still, an opposing theme of superhuman technology also emerged repeatedly during the extrapolation activity. Workshop participants would describe AI and robotics in “superhuman” terms, illustrating the idea that artificially intelligent agents can be used to transcend human memory and capacity limitations. In this section, we describe several ways of limitation transcendence.

The first way of superhuman transcendence is related to surpassing human *physical* capabilities. Robots have been proposed to enhance human capabilities (Vatsal and Hoffman, 2018). WP3 (guitarist) comments that “robots can go beyond [the] physical limitations of what [humans] can do, in precision or stamina.” For example, “it can control airflow much better than a human can.” Another example is “the concept of deliberate practice [for] 10,000 hour, AI and robotics could do this much faster because they [do not] need to spend 10,000 hour [learning] how to play.” Currently, “instruments are designed for ten fingers and within reach of a human arm span, but instruments no longer have to be designed with those constraints” (WP2, percussionist). Incorporating superhuman robots in improvisation could introduce new patterns of playing and new ways of improvising beyond human physical capability, as illustrated, for example, in Weinberg’s work on super-human musical robots (Bretan et al., 2016; Weinberg et al., 2020).

AI and robotics could also overcome the cognitive load limitations that occur when improvising. WP13 (CIS student) liked “the idea of collapsing the space between intent and action” using the fact that computers are “super fast.” WP3 (guitarist) noted that a model could “explore more of a nonhuman approach to learning improvisation, which leads to […] concepts” that humans may not see as quickly or as obviously. Human memory has a limited capacity to store all improvisation genres, but this would not be a difficult task for an AI system. WP2 (percussionist) explains that “a computer [has] a really good memory bank […]. We could feed [it] all music, all music from all parts of Africa, all time periods with whatever is documented.”

Physical-cognitive crossover was also mentioned. WP4 (flutist) speculates a future feature where artificially intelligent improvisational agents “program things like different aesthetic brains so that we can have a corporeal experience of different sets of values.” Agents would assist in “building empathy for other humans […] opening up whole realms of performance practices of the past [and] perhaps the future from other places in the world.” In this form of embodiment, agents provide human improvisers with an immersive bodily experience where embodied systems are connected to their environment beyond physical forces (Ziemke, 2001). In this space, “humans [interact] with robots [in] an artistic sense” or “robots [play] with [other] robots” (WP13, music student).

Not all participants agreed with the superhuman technology theme. Some pointed out limitations to what AI and robotics might contribute to improvisation. The act of improvisation is grounded in the human experience, which may not be replicated artificially. When asked the question, “What can AI and robotics learn by how improvisers think about time, space, actions, and decisions?” musicians noted the gaps to human-level improvisation that AI would need to overcome. One workshop participant stated that “we should teach [or] try to teach AI and robotics to be empathetic and to understand social cues.” For example, “when to play and [when] not to play” or “when to begin or end a piece” (WP2, percussionist). WP4 (flutist) adds that “AI and robotics could learn different sets of cultural norms [..] and how to deviate from those norms.” In this case, “improvisation for AI can be a good way of understanding human-centric concepts” (WP3, guitarist).

Some participants outright questioned the feasibility of teaching AI and robotics aspects of human improvisation. In response to the question of whether you could create improvising AI, WP2 (percussionist) protested that quite possibly “the answer could be, no.” Moreover, the mastery of improvisation requires vulnerability. Improvisation as vulnerability is the ability to open oneself to risk. WP12 (design student) expressed that “there is a very important lesson that we can learn from our vulnerability, which is usually something that we do not see when we talk about machines and [AI]. We see them as really powerful tools.” The hesitations described above indicate that the promise of an AI or robotic superhuman improviser comes with a caveat.

### 7 FOLLOW-UP WORKSHOPS

The knowledge of the musical performers dominated the discussion in the first workshop. To further understand how ME and CIS students conceptualize improvisation, we conducted two shorter follow-up workshops based on insights from the first workshop. We recruited a group of ME and CIS graduate students in robotics labs across the authors’ university to participate in the follow-up workshops. Follow-up workshops were informal collaborative sessions that occurred at two robotics labs at our university across two campuses. The workshops occurred on separate dates, each lasting 2 hour. The first follow-up session consisted of two graduate students in ME and CIS departments. There were four workshop participants in the second follow-up workshop: two graduate students, one post-doctoral fellow, and one faculty member in CIS. None of the follow-up workshop participants attended the first joint workshop with the musicians. We recorded one of the sessions with a total of 101 minutes of audio captured in one fragment. In the transcription, we assigned tags at time points that corresponded to each theme. Whereas our initial workshop focused on learning about the musicians’ improvisational experiences, the focus of the follow up sessions was to understand more deeply how improvisation applies to AI and robotics.

We began the follow-up by again asking the question: “What is an improvising musical robot?” We gave everyone 5 minutes to write down ideas. Ideas ranged from a “random music player,” to a “robot that dances to music,” to a “listening box” with big ears that helps a human improviser listen to music.

We then presented a preliminary version of the above-listed themes of improvisation (in Sections 4 And 6) and led additional discussion sessions. During the sessions, we uncovered more detailed nuances and hesitations about the “translations” of the concepts into machine learning and artificial intelligence terms beyond the findings from the first joint workshop. Improvisation as Assistance was further developed into three sub-themes: Improvisation as a *Design Process*, Improvisation as *Collective Intelligence*, and Improvisation as *Evaluation*.

### 7.1 Improvisation as Randomness

Randomness was again discussed as a promising way to incorporate AI and robotics in musical improvisation. Randomness specifically tied back to the loss of control that is inherent to improvisation. Building on the notion of “adaptability” as the ability to be influenced by one’s surrounding environment while performing, a robotic system might act as an external force causing random disturbances during a performance.

Some suggested specific ideas of how a random AI system could be incorporated into improvisation. One route would be for it to “add machine noise” or “random sounds to a music piece” to make the music “sound like something else” (WP18, ME student). Building on this idea, WP17 (CIS student) imagined an improvising robot that takes “a song and inserts random pauses [or] notes.” It could also do “unexpected things” outside of cultural norms, for example (WP17, CIS student). This random element in performance encourages a human improviser to rely less on previously learned material and generate solutions on the spot.

### 7.2 Improvisation as Assistance

In the follow-up workshops, much of the conversation revolved around the role of AI and robotics in assisting the decision-making process and providing feedback for a human improviser. This role was mainly conceptualized by framing the improvisation process around three process-oriented themes: design as a metaphor for improvisation, collective intelligence, and methods for evaluating improvisational elements.

#### 7.2.1 Improvisation as a Design Process

In the second follow-up workshop, the double-diamond design process was used to model the divergent and convergent processes of generating new sound material (British Design Council, 2007). In the divergent phase, where ideas are generated, an AI system could take an initial idea or source of inspiration from a human improviser and create many configurations for an improvisational piece. On the convergent side of the process, where ideas are filtered into concrete concepts, an AI system can act as a filter, reviewing ideas from the previous state and giving recommendations for the next steps. The model could parse contextual data to help an improviser make decisions in the exact moment, monitor each musical improvisation session, and suggest areas of improvement, such as tone, pitch, and timbre. For this system to work, there must be a set standard for elimination or deciding where to go next. Some parameters might be the level of originality, fluency, pitch, or tone.

#### 7.2.2 Improvisation as Collective Intelligence

A second way AI could assist a human improviser would be by viewing the collaboration between humans and technology as collective intelligence. WP20 (CIS student) describes a continuous transition between humans playing “music as we understand it” and “mostly noise” generated by algorithms. Here, humans would be at the beginning and end “nodes” of the diamond (mentioned earlier in the design process), and agents would be situated in the middle between the divergent and convergent sides of sound creation. The agents would draw “inspiration from any music that ever existed,” and then a human improviser can select material to work into the piece. The agent could guide by “moving forward into a direction [that is] maybe not obvious to you,” like a “blind spot detector” for music (WP20, CIS student). WP22 (CIS faculty) suggested the concept of an ecology of agents. Each agent has a different role in the sound creation process. One “agent that creates lots of ideas” and another that “picks some amplifies some.” This ecology forms a collective intelligence that “takes away the pressure on any one of them to be perfect” (WP20, CIS student).

#### 7.2.3 Improvisation as Evaluation

Improvisation as Evaluation is about using AI and robotics to provide a feedback mechanism for improvisation. A recurring theme from both follow-up workshops is the challenge of setting a standard for “good improvisation.” We observed that it was challenging for most follow-up workshop participants to define a measure of accuracy, and specifically, deciding who or what gets to judge what is competent in improvisation. Nonetheless, an evaluation function might be based on “expert [or] audience” judgment, and some parameters could be “originality, completeness, fluency, [and] impact (reward)” (WP18, ME student). A potential model might read the audience or expert critic’s facial expressions, gestures, output, and provide a score for improvisational performance. Evaluation can also come in the form of an AI instrument contributing to the music by generating sound output. Using an evaluation metric, explicitly set or learned implicitly, it could anticipate what the musical piece needs by “building and adding” to the music currently played and “chang[ing] and tweak[ing] things gracefully” (WP17, CIS student).

### 7.3 Improvisation as Data

ME and CIS students mostly viewed sound as input data into a machine-learning model. WP18 (ME student) noted that “music [is] data,” so music files can be fed into a model that can “extract shared elements [or] patterns” thus, the music acts as “previously learned materials” in the system. In the transformation process, it can “edit some elements of the music” including the “beat, rhythm, [or] pause.” The focus of these discussions was on the process of importing sound data into a model and extracting patterns. Unlike conversations from the previous joint workshop, there was no mention of using instruments as data collection devices.

### 7.4 Robots as “Superhumans”

Finally, it is worth noting that, in the follow-up workshops made up primarily of ME and CIS students, we discovered that the thought of robots transcending the limitations of human memory and physical capabilities was met with skepticism. This is a useful contrast to our findings from the joint workshop, driven by the musical performers. However, while the musicians highlighted the more nuanced human traits, such as vulnerability and life experience as obstacles for AI improvisers, engineering and CIS students focused on the limitations of technology.

For example, WP17 (CIS student) commented that robots are often unable to “do what humans do easily.” WP17 (CIS student) focused on ways in which AI and robotics are still limited to physical constraints: “it [can not] defy gravity,” or “it can not be in two places at once.” Others chose to focus on the skills that robots can do well”storage, calculation, and computation. Instead of building a “superhuman,” one might leverage the fact that AI and robotics can be used as a “memory” bank for human improvisers, and can aid in the “transformation and synthesis” of new sound creation (WP18, ME student).

## 8 Discussion and Design Considerations

We discovered interesting connections between AI, robotics, and improvisation through bringing together musicians specializing in improvised performances with ME and CIS students in a series of design workshops. In our first workshop, four themes emerged as the musicians unpacked improvisation into its fundamental components. The first theme emphasized spontaneity, which is the ability to connect thinking and acting, compressing time, and making decisions on the spot. The second spoke of adaptability, responding to the environment, but even more so, to other improvisers. The third theme was learning, which builds on experience and knowledge, integrating the many years of skill-acquisition with the ability to act in the moment. Finally, musical improvisers emphasized the existence of an inner voice that guided their playing.

The workshop was framed to all participants to gain knowledge toward building improvising robots or AI-enabled musical instruments. In this context, the discussion did not remain in the musical realm alone, and all participants attempted to translate the complex structure of improvisation into engineering concepts. An analysis of the discussions surrounding building such systems reflected three of the above-mentioned four themes, albeit in diminished form. AI and robotic improvisers were imagined to use and provide *randomness*, would make use of *data*, and were suggested to *assist* the human improvisers. These three themes can be mapped to the notions of spontaneity, learning, and mutual adaptation (Figure 3). However, randomness must be viewed as a reduced form of spontaneity. Data and machine learning are an impoverished metaphors for human learning, especially for the cognitive-embodied kind required for an instrument. Similarly, assistance is a one-directional and subservient version of the rich back-and-forth that musicians provide in an improvised performance. The notion of an inner voice was completely missing from the discussion of AI and robot improvisers in all workshops.

What can designers of improvising robots and other AI-supported musical machines learn from the tension uncovered in our workshops? We present several considerations for design based on the themes and findings listed above.

### 8.1 Minding the Gap

When engineers think of improvisation, they run the risk of centering around technical concepts, such as randomness, data, and technology that assists a human musician. We recommend considering the gap between these notions and the richer ones brought forth by musicians: spontaneity, learning, and adaptability.

When a roboticist thinks of adding randomness to a machine improvisation process, they may instead consider to model spontaneity. Spontaneity affords one the ability to generate new and surprising ideas in real-time instead of limiting oneself to mathematical predeterminants or pseudo-random processes. Are random processes surprising at all? Do they capture the sense of “now” that spontaneity implies? Is randomness the contraction of the past, present, and future? We recommend designers of improvising robots to spend time sketching out a possible path from randomness to spontaneity when building improvising systems.

Similarly, robotic musical improvisation should be more than a functional output of sound data. The method of extracting patterns from data files is a flattened version of the learning processes underlying the ability to improvise. We encourage designers to explore the value to be gained from a more holistic approach to learning to produce sound and gestures. Our findings suggest an expanded notion of machine learning to include compositional, experiential, and contextual modes of learning.

Third, improvisation as assistive technology can fall short of the openness implied by the mutual adaptation in human improvisation. An improvising robot may be able to do more than assist a human in their exploration. We invite designers to ask: how can a robot contribute to sound creation while influencing and being influenced by its surrounding environment? Roboticists can use their designs to highlight this gap to consider power dynamics and calibrate user expectations around control and autonomy, much like previous interactive computer-based music models facilitated communication between performers, audience members, and instruments. For example, the works by George Lewis have established networks of non-hierarchical relationships between humans and nonhuman devices and between humans and humans to challenge institutional authorities (Lewis, 2017).

Finally, robot designers should ask: what is a robot’s inner voice? Can one imagine the proverbial “baggage” that the robot brings to the performance? How does it interact with the other three themes, which were more natural for engineers to consider?

### 8.2 Superhuman, but Not Good Enough

Although robot improvisation was described in diminished terms compared to human improvisation, participants in the workshop often landed on the idea that AI and robotics could be superhuman, whether by overcoming the physical or the mental limitations of humans. The idea of robots overcoming human limitations is also a common theme in the robotics literature (Van Den Berg et al., 2010), while some have mounted a scholarly critique of robotics and AI as superhuman (Haslam et al., 2008). Why do participants view robotic improvisers as superhuman, given the above analysis that shows that the imagined roles for technology fall short of those identified for human improvisers? One may argue that the superhuman abilities that participants imagine are limited to rote physical and memory-related tasks. Be it as it may, we note the tension between the technological promise that machines may outdo humans in the task of improvisation and the lack of core competencies required of an improvising agent that machines can provide.

When questioning the possibility of a superhuman robotic improviser, musicians and roboticists listed very different rationalizations for their skepticism. Musicians and designers emphasized subtle aspects of humanness in improvisation, such as life experience, standards, vulnerability, empathy, and risk. Engineering and CIS students emphasized mostly technological limitations. The different rationales also highlight the diverging vocabulary of the two communities we worked with and emphasizes the need for a translative effort to collaborate between these two populations.

Future research in AI-mediated and robotic improvisation should make their artifact’s relationship to the superhuman theme explicit. A robot may employ superhuman capabilities, such as computational power, to surpass human memory and physical capabilities when improvising. But roboticists must embrace and highlight the ways in which a robotic improviser falls short in their design. The tension between utopia and disappointment will enrich the expressive potential of a human-robot joint improvisation.

### 8.3 Fragility and Uncertainty as Metrics for Success

We also argue for a new paradigm in building AI and robotic models by embedding improvisation principles in the conception phase, as well as in the metrics for their evaluation. AI and robotics engineers usually measure their work with respect to metrics of stability, reliability, and performance (in the engineering sense). One of the gaps exposed in our analysis of the superhuman theme is that empathy, vulnerability, and risk are at the core of good improvisation.

Designers of improvising robots should imagine artificial improvising models that embrace uncertainty and fragility. In this alternative scenario, models are built under ambiguous and incomplete conditions that produce fluid and temporal systems. Models evolve into new creations where new knowledge is produced in real-time. Here, code might break, or robots might consist of collapsible or decaying components. A musician might have to build an instrument as they play on a stage. Also, roboticists must learn to be comfortable relinquishing some control since their creation might be used in a different manner than intended.

In summary, the convergence of experts from divergent fields could help roboticists and musicians who want to collaborate on building improvising machines make sense of both the promises and the gaps toward this goal. The qualitative exploration provided here could help guide toward productive themes to explore and warn of potential pitfalls in the translation of concepts between the performance and engineering communities. When examining all of the gaps mentioned above, it is also important that both communities should make explicit their delimited views of improvisation.

## 9 Limitations

The themes and insights provided above are subject to several methodological limitations. First, the workshops were not made up of a representative sample of musicians or researchers, but each was an organized research activity between existing collaborators. Along the same lines, the authors of this paper served in multiple roles: we were organizers of the workshops and active participants in the discussions. As a result, this paper’s thematic analysis was not done blindly or by multiple, independent, and correlated coders. Instead, the analysis came out of a discussion of the insights gleaned from the documentation collected during the workshops. Subsequently, the work described here falls under the category of qualitative conclusions drawn from an embedded research activity, rather than a controlled study of music improvisation.

## Data Availability

The raw data supporting the conclusions of this article will be made available by the authors, without undue reservation.
